# Supplementary material for: Characteristics and incidence trends of adults hospitalized with community-acquired pneumonia in Portugal, pre-pandemic
Source: PLoS One. 2025 May 16;20(5):e0322623. doi: 10.1371/journal.pone.0322623 (PMC12084036; doi:10.1371/journal.pone.0322623)
Supplement: S1 Table — (DOCX) [file pone.0322623.s001.docx]

**Title: Characteristics and incidence trends of adults hospitalized with community-acquired pneumonia in Portugal, pre-pandemic**

**Supplementary material**

S1 Table. List of ICD-9-CM and ICD-10-CM codes

| **Code** | **Description** |
| --- | --- |
| **ICD-9-CM** |  |
| 480 | Viral pneumonia |
| 481 | Pneumococcal pneumonia [Streptococcus pneumoniae pneumonia] |
| 482 | Other bacterial pneumonia |
| 483 | Pneumonia due to other specified organism |
| 484 | Pneumonia in infectious diseases classified elsewhere |
| 485 | Bronchopneumonia, organism unspecified |
| 486. | Pneumonia, organism unspecified |
| 487.0 | Influenza with pneumonia |
| **ICD-10-CM** |  |
| J09.X1 | Influenza due to identified novel influenza A virus with pneumonia |
| J10.00 | Influenza due to other identified influenza virus with unspecified type of pneumonia |
| J10.01 | Influenza due to other identified influenza virus with the same other identified influenza virus pneumonia |
| J10.08 | Influenza due to other identified influenza virus with other specified pneumonia |
| J11.00 | Influenza due to unidentified influenza virus with unspecified type of pneumonia |
| J11.08 | Influenza due to unidentified influenza virus with specified pneumonia |
| J12 | Viral pneumonia, not elsewhere classified |
| J13 | Pneumonia due to Streptococcus pneumoniae |
| J14 | Pneumonia due to Hemophilus influenzae |
| J15 | Bacterial pneumonia, not elsewhere classified |
| J16 | Pneumonia due to other infectious organisms, not elsewhere classified |
| J17 | Pneumonia in diseases classified elsewhere |
| J18 | Pneumonia, unspecified organism |

References: List of ICD-9-CM codes adapted from Froes et al. (2013)[18]. List of ICD-10-CM codes adapted from Lewnard et al. (2022)[19]. Codes listed encompass all codes included.
