# Supplementary material for: Characteristics and incidence trends of adults hospitalized with community-acquired pneumonia in Portugal, pre-pandemic
Source: PLoS One. 2025 May 16;20(5):e0322623. doi: 10.1371/journal.pone.0322623 (PMC12084036; doi:10.1371/journal.pone.0322623)
Supplement: S2 Table — (DOCX) [file pone.0322623.s002.docx]

**Title: Characteristics and incidence trends of adults hospitalized with community-acquired pneumonia in Portugal, pre-pandemic**

**Supplementary material**

S2 Table. ICD-9-CM and ICD-10-CM Coding Algorithms for Elixhauser Comorbidities Index[24]

| **Comorbidities** | **Enhanced ICD-9-CM** | **ICD-10-CM** |
| --- | --- | --- |
| Congestive heart failure | 398.91, 402.01, 402.11, 402.91, 404.01, 404.03, 404.11, 404.13, 404.91, 404.93, 425.4-425.9, 428.x | I09.9, I11.0, I13.0, I13.2, I25.5, I42.0, 142.5-I42.9, I43.x, I50.x, P29.0 |
| Peripheral vascular disorders | 093.0, 437.3, 440.x, 441.x, 443.1- 443.9, 447.1, 557.1, 557.9, V43.4 | I70.x, I71.x, I73.1, I73.8, I73.9, I77.1, I79.0, I79.2, K55.1, K55.8, K55.9, Z95.8, Z95.9 |
| Chronic pulmonary disease | 416.8, 416.9, 490.x -505.x, 506.4, 508.1, 508.8 | I27.8, 127.9, J40.x-J47.x, J60.x-J67.x, J68.4, J70.1, J70.3 |
| Diabetes | 250.0-250.3, 250.4-250.9 | E10.0, E10.1, E10.9, E11.0, E11.1, E11.9, E12.0, E12.1, E12.9, E13.0, E13.1, E13.9, E14.0, E14.1, E14.9, E10.2-E10.8, E11.2-E11.8, E12.2- E12.8, E13.2-E13.8, E14.2-E14.8 |
| Chronic renal disease | 403.01, 403.11, 403.91, 404.02, 404.03, 404.12, 404.13, 404.92, 404.93, 585.x, 586.x, 588.0, V42.0, V45.1, V56.x | I12.0, I13.1, N18.x, NI9.x, N25.0, Z49.0-Z49.2, Z94.0, Z 1 99.2 |
| Liver disease | 070.22, 070.23, 070.32, 070.33, 070.44, 070.54, 070.6, 070.9, 456.0-456.2, 570.x, 571.x, 572.2-572.8, 573.3, 573.4, 573.8, 573.9, V42.7 | B18.x, I85.x, I86.4, I98.2, K70.x, K71.1, K71.3- K71.5, K71.7, K72.xK74.x, K76.0, K76.2- K76.9. Z94.4 |
| HIV/AIDS | 042.x-044.x | B20.x-B22.x, B24.x |
| Metastatic cancer | 196.x-199.x | C77.x-C80.x |
| Solid tumour without metastasis | 140.x-172.x, 174.x-195.x | C00.x-C26.x, C30.x-C34.x, C37.x-C41.x, C43.x, C45.x-C58.x, C60.x-C76.x, C97.x |
| Rheumatoid arthritis/ collagen vascular diseases | 446.x, 701.0, 710.0-710.4, 710.8, 710.9, 711.2, 714.x, 719.3, 720.x, 725.x, 728.5, 728.89, 729.30 | L94.0, L94.1, L94.3, M05.x, M06.x, M08.x, M12.0, M12.3, M30.x, M31.0-M31.3, M32.x-M35.x, M45.x, M46.1, M46.8, M46.9 |
