# Supplementary material for: Characteristics and incidence trends of adults hospitalized with community-acquired pneumonia in Portugal, pre-pandemic
Source: PLoS One. 2025 May 16;20(5):e0322623. doi: 10.1371/journal.pone.0322623 (PMC12084036; doi:10.1371/journal.pone.0322623)
Supplement: S3 Table — (DOCX) [file pone.0322623.s003.docx]

**Title: Characteristics and incidence trends of adults hospitalized with community-acquired pneumonia in Portugal, pre-pandemic**

**Supplementary material**

S3 Table. Criteria for estimating number of adults living with comorbidities

|  | **Comorbidity** | **Definition** | | **Period** | | **Geographical area** | | **Age group** | |
| --- | --- | --- | --- | --- | --- | --- | --- | --- | --- |
|  |  | **Hospitalizations data** | **Prevalence data** | **Hospitalizations data** | **Prevalence data** | **Hospitalizations data** | **Prevalence data** | **Hospitalizations data** | **Prevalence data** |
| **1** | **Peripheral vascular disorders** | Peripheral vascular disorders | Peripheral artery disease | 2010-2018 | | Mainland Portugal | Portugal | ≥18 | ≥20 |
|  | **HIV/AIDS** | HIV/AIDS | HIV/AIDS | 2010-2018 | | Mainland Portugal | Portugal | ≥18 | ≥20 |
|  | **Rheumatoid arthritis/ collagen vascular diseases** | Rheumatic/inflammatory disease | Rheumatoid Arthritis | 2010-2018 | | Mainland Portugal | Portugal | ≥18 | ≥20 |
|  | **Liver disease** | Moderate/Severe Liver Disease | Cirrhosis and other chronic liver diseases | 2010-2018 | | Mainland Portugal | Portugal | ≥18 | ≥20 |
| **2** | **Solid tumor without metastasis** | Solid tumor without metastasis | Total cancer | 2010-2018 | | Mainland Portugal | Portugal | ≥18 | ≥20 |
|  | **Metastatic cancer** | Metastatic cancer | Total cancer | 2010-2018 | | Mainland Portugal | Portugal | ≥18 | ≥20 |
| **3** | **Complicated and Uncomplicated Diabetes** | Complicated and Uncomplicated Diabetes | Diabetes | 2010-2018 | 2005/2006 | Mainland Portugal | Mainland Portugal | ≥18 | ≥15 |
|  |  |  |  | 2010-2018 | 2014 | Mainland Portugal |  | ≥18 |  |
|  |  |  |  | 2010-2018 | 2019 | Mainland Portugal |  | ≥18 |  |
| **4** | **Chronic renal disease/ Renal Failure** | Chronic renal disease | Renal insufficiency | 2010-2018 | 2005/2006 | Mainland Portugal | Mainland Portugal | ≥18 | ≥15 |
|  |  |  | Renal problems | 2010-2018 | 2014 | Mainland Portugal |  | ≥18 |  |
|  |  |  |  | 2010-2018 | 2019 | Mainland Portugal |  | ≥18 |  |
|  | **Chronic Pulmonary disease** | Chronic Pulmonary Disease | Chronic bronchitis or emphysema | 2010-2018 | 2005/2006 | Mainland Portugal | Mainland Portugal | ≥18 | ≥15 |
|  |  |  | Chronic bronchitis, COPD or emphysema | 2010-2018 | 2014 | Mainland Portugal |  | ≥18 |  |
|  |  |  | Chronic bronchitis, COPD or emphysema | 2010-2018 | 2019 | Mainland Portugal |  | ≥18 |  |
| **5** | **Congestive heart failure** | Congestive heart failure | Heart Failure | 2010-2018 | 2011 | Mainland Portugal | Portugal | ≥18 | ≥25 |
|  |  |  |  |  | 2018 |  |  |  |  |

Note: Peripheral vascular disorders, HIV/AIDS, rheumatoid arthritis/collagen vascular diseases, liver disease, solid tumor without metastasis and metastatic cancer prevalence were extracted from IHME (2010-18)[14]. Complicated and uncomplicated diabetes, chronic renal disease/renal failure and chronic pulmonary disease prevalence were extracted from National Health Survey (2005/2006, 2014, 2019)[13]. Congestive heart failure prevalence was extracted from EPICA (2011 and 2018)[15,16].
