# Supplementary material for: Characteristics and incidence trends of adults hospitalized with community-acquired pneumonia in Portugal, pre-pandemic
Source: PLoS One. 2025 May 16;20(5):e0322623. doi: 10.1371/journal.pone.0322623 (PMC12084036; doi:10.1371/journal.pone.0322623)
Supplement: S4 Table — (DOCX) [file pone.0322623.s004.docx]

**Title: Characteristics and incidence trends of adults hospitalized with community-acquired pneumonia in Portugal, pre-pandemic**

**Supplementary material**

S4 Table. Number of CAP hospitalizations and number of inhabitants, estimated for each year under study, total and by sex and age group, 2010-18

|  | **Year** | | | | | | | | |
| --- | --- | --- | --- | --- | --- | --- | --- | --- | --- |
|  | **2010** | **2011** | **2012** | **2013** | **2014** | **2015** | **2016** | **2017** | **2018** |
| **Sex** |  |  |  |  |  |  |  |  |  |
| **Male** |  |  |  |  |  |  |  |  |  |
| Number of CAP hospitalizations | 30652 | 31240 | 31976 | 28983 | 27535 | 28514 | 27472 | 26910 | 24552 |
| Number of inhabitants | 3 874 767 | 3 867 297 | 3 843 261 | 3 820 406 | 3 806 055 | 3 804 518 | 3 805 896 | 3 814 561 | 3 826 060 |
| **Female** |  |  |  |  |  |  |  |  |  |
| Number of CAP hospitalizations | 23905 | 24814 | 26448 | 23113 | 22693 | 24381 | 23084 | 22915 | 21358 |
| Number of inhabitants | 4 364 932 | 4 373 810 | 4 364 593 | 4 356 395 | 4 353 429 | 4 355 087 | 4 354 766 | 4 364 514 | 4 379 135 |
| **Age group** |  |  |  |  |  |  |  |  |  |
| **18-29** |  |  |  |  |  |  |  |  |  |
| Number of CAP hospitalizations | 936 | 991 | 1229 | 508 | 608 | 542 | 545 | 441 | 457 |
| Number of inhabitants | 1 394 117 | 1 359 674 | 1 316 904 | 1 281 403 | 1 254 753 | 1 244 530 | 1 237 822 | 1 240 986 | 1 247 767 |
| **30-49** |  |  |  |  |  |  |  |  |  |
| Number of CAP hospitalizations | 4205 | 4147 | 3671 | 2987 | 3109 | 2786 | 2735 | 2307 | 2277 |
| Number of inhabitants | 3 003 834 | 2 973 554 | 2 937 922 | 2 896 098 | 2 851 615 | 2 808 238 | 2 763 813 | 2 724 291 | 2 687 870 |
| **50-64** |  |  |  |  |  |  |  |  |  |
| Number of CAP hospitalizations | 6548 | 7138 | 6493 | 6062 | 5855 | 5752 | 5994 | 5405 | 5104 |
| Number of inhabitants | 1 934 806 | 1 955 590 | 1 976 818 | 1 985 497 | 2 002 466 | 2 018 305 | 2 030 970 | 2 042 403 | 2 056 669 |
| **65-74** |  |  |  |  |  |  |  |  |  |
| Number of CAP hospitalizations | 9374 | 9410 | 9333 | 8162 | 7841 | 7934 | 7723 | 7629 | 7034 |
| Number of inhabitants | 991 663 | 991 766 | 997 990 | 1 015 577 | 1 031 883 | 1 055 317 | 1 083 392 | 1 111 851 | 1 134 068 |
| **75-84** |  |  |  |  |  |  |  |  |  |
| Number of CAP hospitalizations | 18965 | 19054 | 20087 | 17793 | 16719 | 17929 | 16437 | 16056 | 14305 |
| Number of inhabitants | 696 979 | 715 769 | 724 264 | 731 983 | 739 930 | 743 630 | 743 857 | 746 896 | 756 618 |
| **≥85** |  |  |  |  |  |  |  |  |  |
| Number of CAP hospitalizations | 14529 | 15314 | 17611 | 16584 | 16096 | 17952 | 17122 | 17987 | 16733 |
| Number of inhabitants | 218 300 | 244 754 | 253 956 | 266 243 | 278 837 | 289 585 | 300 808 | 312 648 | 322 203 |
