# Supplementary material for: Characteristics and incidence trends of adults hospitalized with community-acquired pneumonia in Portugal, pre-pandemic
Source: PLoS One. 2025 May 16;20(5):e0322623. doi: 10.1371/journal.pone.0322623 (PMC12084036; doi:10.1371/journal.pone.0322623)
Supplement: S5 Table — (DOCX) [file pone.0322623.s005.docx]

**Title: Characteristics and incidence trends of adults hospitalized with community-acquired pneumonia in Portugal, pre-pandemic**

**Supplementary material**

S5 Table. Number of CAP hospitalizations and number of adults living with the comorbidity, estimated for each year, by comorbidity, 2010-18

| **Comorbidities** | **Year** | | | | | | | | |
| --- | --- | --- | --- | --- | --- | --- | --- | --- | --- |
|  | **2010** | **2011** | **2012** | **2013** | **2014** | **2015** | **2016** | **2017** | **2018** |
| **Chronic pulmonary disease** |  |  |  |  |  |  |  |  |  |
| Number of CAP hospitalizations | 8699 | 9670 | 9439 | 10387 | 10471 | 11520 | 10853 | 9439 | 9047 |
| Number of people living with the comorbidity | 534892 | 529596 | 524299 | 519003 | 513706 | 508409 | 503113 | 497816 | 492520 |
| **Congestive heart failure** |  |  |  |  |  |  |  |  |  |
| Number of CAP hospitalizations | 12122 | 12955 | 14983 | 13710 | 13416 | 14686 | 13871 | 14983 | 14251 |
| Number of people living with the comorbidity | 367472 | 371264 | 375056 | 378847 | 382639 | 386430 | 390222 | 394013 | 397805 |
| **Peripheral heart disorder** |  |  |  |  |  |  |  |  |  |
| Number of CAP hospitalizations | 1398 | 1596 | 1600 | 1920 | 1795 | 2005 | 1836 | 1600 | 1465 |
| Number of people living with the comorbidity | 406584 | 412931 | 418785 | 425094 | 431660 | 438090 | 443368 | 448308 | 456774 |
| **Diabetes** |  |  |  |  |  |  |  |  |  |
| Number of CAP hospitalizations | 12390 | 12971 | 13540 | 13440 | 13120 | 13988 | 13618 | 13540 | 12687 |
| Number of people living with the comorbidity | 742544 | 763825 | 785105 | 806386 | 827666 | 838836 | 850006 | 861177 | 872347 |
| **Rheumatoid arthritis/collagen vascular diseases** |  |  |  |  |  |  |  |  |  |
| Number of CAP hospitalizations | 798 | 844 | 827 | 871 | 930 | 992 | 1006 | 827 | 805 |
| Number of people living with the comorbidity | 35131 | 35678 | 36272 | 36856 | 37348 | 37709 | 37536 | 37345 | 37619 |
| **HIV/AIDS** |  |  |  |  |  |  |  |  |  |
| Number of CAP hospitalizations | 253 | 259 | 247 | 251 | 184 | 225 | 198 | 247 | 253 |
| Number of people living with the comorbidity | 30711 | 30967 | 31356 | 31911 | 32740 | 33812 | 35032 | 36353 | 37718 |
| **Solid tumor without metastasis and Mestastatic cancer** |  |  |  |  |  |  |  |  |  |
| Number of CAP hospitalizations | 3853 | 4077 | 4431 | 4231 | 4033 | 4503 | 4382 | 4431 | 3848 |
| Number of people living with the comorbidity | 305407 | 309651 | 312497 | 316462 | 321061 | 324622 | 334373 | 342848 | 348149 |
| **Liver disease** |  |  |  |  |  |  |  |  |  |
| Number of CAP hospitalizations | 2512 | 2643 | 2232 | 2538 | 2606 | 2613 | 2705 | 2232 | 2113 |
| Number of people living with the comorbidity | 1943182 | 1959969 | 1976945 | 1993541 | 2008124 | 2019480 | 2025360 | 2030698 | 2040340 |
| **Chronic renal disease** |  |  |  |  |  |  |  |  |  |
| Number of CAP hospitalizations | 6316 | 7331 | 9219 | 8152 | 8377 | 9448 | 8728 | 9219 | 8431 |
| Number of people living with the comorbidity | 430258 | 424308 | 418359 | 412409 | 406460 | 400511 | 394561 | 388612 | 382662 |
