# Supplementary material for: Characteristics and incidence trends of adults hospitalized with community-acquired pneumonia in Portugal, pre-pandemic
Source: PLoS One. 2025 May 16;20(5):e0322623. doi: 10.1371/journal.pone.0322623 (PMC12084036; doi:10.1371/journal.pone.0322623)
Supplement: S6 Table — (DOCX) [file pone.0322623.s006.docx]

**Title: Characteristics and incidence trends of adults hospitalized with community-acquired pneumonia in Portugal, pre-pandemic**

**Supplementary material**

S6 Table. Sensitivity analysis: Incidence rates of CAP hospitalizations per 1,000 inhabitants, estimated for each year, by sex and age group, 2013-18

|  | **Incidence rates (95%CI)** **per 1,000 inhabitants** | | | | | |
| --- | --- | --- | --- | --- | --- | --- |
|  | **2013** | **2014** | **2015** | **2016** | **2017** | **2018** |
| **Sex** |  |  |  |  |  |  |
| Male | 7.5 (7.4 - 7.6) | 7.2 (7.2 - 7.3) | 7.5 (7.4 - 7.6) | 7.2 (7.1 - 7.3) | 7.1 (7.0 - 7.1) | 6.4 (6.3 - 6.5) |
| Female | 5.3 (5.2 - 5.3) | 5.2 (5.2 - 5.3) | 5.6 (5.5 - 5.7) | 5.3 (5.2 - 5.4) | 5.3 (5.2 - 5.3) | 4.9 (4.9 - 5.0) |
| **Age** **group (years)** |  |  |  |  |  |  |
| 18-29 | 0.4 (0.4 - 0.4) | 0.5 (0.4 - 0.5) | 0.4 (0.4 - 0.5) | 0.4 (0.4 - 0.5) | 0.4 (0.3 - 0.4) | 0.4 (0.3 - 0.4) |
| 30-49 | 1.0 (1.0 - 1.1) | 1.1 (1.1 - 1.1) | 1.0 (1.0 - 1.0) | 1.0 (1.0 – 1.0) | 0.8 (0.8 - 0.9) | 0.8 (0.8 - 0.9) |
| 50-64 | 3.0 (2.9 - 3.1) | 2.9 (2.9 - 3.0) | 2.9 (2.8 - 2.9) | 3.0 (2.9 – 3.0) | 2.6 (2.6 - 2.7) | 2.5 (2.4 - 2.6) |
| 65-74 | 7.9 (7.7 - 8.1) | 7.6 (7.4 - 7.8) | 7.5 (7.4 - 7.7) | 7.1 (6.0 - 7.3) | 6.9 (6.7 - 7.0) | 6.2 (6.1 - 6.3) |
| 75-84 | 24.0 (23.7 - 24.4) | 22.6 (22.3 - 22.9) | 24.1 (23.8 - 24.5) | 22.1 (21.8 - 22.5) | 21.5 (21.2 - 21.8) | 18.9 (18.6 - 19.2) |
| ≥85 | 62.0 (61.0 - 62.9) | 57.8 (56.9 - 58.7) | 62.0 (61.1 - 62.9) | 57.0 (56.1 - 57.8) | 57.6 (56.7 - 58.4) | 52.0 (51.2 - 52.8) |

Note: The sensitivity analysis considered the same events as the main analysis, but added the admissions for which CAP was recorded as additional diagnosis and POA was coded as unknown or undetermined. Because the POA flag was implemented in Portugal only in 2013, 2010-2012 could not be included.

CI: confidence interval.
