# Supplementary material for: Characteristics and incidence trends of adults hospitalized with community-acquired pneumonia in Portugal, pre-pandemic
Source: PLoS One. 2025 May 16;20(5):e0322623. doi: 10.1371/journal.pone.0322623 (PMC12084036; doi:10.1371/journal.pone.0322623)
Supplement: S7 Table — (DOCX) [file pone.0322623.s007.docx]

**Title: Characteristics and incidence trends of adults hospitalized with community-acquired pneumonia in Portugal, pre-pandemic**

**Supplementary material**

S7 Table. Sensitivity analysis: Incidence rates of CAP hospitalizations per 1,000 adults living with the comorbidity, estimated for each year, by comorbidity, 2013-18

| **Comorbidities** | **Incidence rates (95%CI)** **per 1,000 people living with the comorbidity** | | | | | |
| --- | --- | --- | --- | --- | --- | --- |
|  | **2013** | **2014** | **2015** | **2016** | **2017** | **2018** |
| Chronic pulmonary disease | 20.0 (19.6 - 20.4) | 20.4 (20.0 - 20.8) | 22.7 (22.3 - 23.1) | 21.6 (21.2- 22.0) | 19.0 (18.6 - 19.4) | 18.4 (18.0 - 18.6) |
| Congestive heart failure | 36.2 (35.6 - 36.8) | 35.2 (34.6 - 35.8) | 38.0 (37.4 - 38.7) | 35.6 (35.0- 36.2) | 38.0 (37.4 - 38.7) | 35.8 (35.3 - 36.4) |
| Peripheral vascular disorders | 4.5 (4.3 - 4.7) | 4.2 (4.0 - 4.4) | 4.6 (4.4 - 4.8) | 4.2 (4.0- 4.3) | 3.6 (3.4 - 3.7) | 3.2 (3.0 - 3.4) |
| Diabetes | 16.7 (16.4 - 17.0) | 15.9 (15.6 - 16.1) | 16.7 (16.4 - 17.0) | 16.0 (15.8- 16.3) | 15.7 (15.5 - 16.0) | 14.5 (14.3 - 14.8) |
| Rheumatoid arthritis/ collagen vascular diseases | 23.6 (22.1 - 25.2) | 24.4 (22.9 - 26.0) | 26.3 (24.7 - 27.9) | 27.5 (25.8- 29.1) | 22.2 (20.7 - 23.7) | 21.4 (19.9 - 22.9) |
| HIV/AIDS | 7.9 (6.9 - 8.9) | 5.6 (4.8 - 6.4) | 6.7 (5.8 - 7.5) | 5.7 (4.9- 6.4) | 6.8 (5.9 - 7.6) | 6.7 (5.9 - 7.5) |
| Solid tumour without metastasis and Mestastatic cancer | 13.4 (13.0 - 13.8) | 12.6 (12.2 - 13.0) | 13.9 (13.5 - 14.3) | 13.1 (12.8- 13.5) | 12.9 (12.6 - 13.3) | 11.1 (10.7 - 11.4) |
| Liver disease | 1.3 (1.2 – 1.3) | 1.3 (1.3 - 1.4) | 1.3 (1.2 - 1.3) | 1.3 (1.3- 1.4) | 1.1 (1.1 - 1.1) | 1.0 (1.0 - 1.1) |
| Chronic renal disease | 19.8 (19.4 - 20.2) | 20.6 (20.2 - 21.1) | 23.6 (23.1 - 24.1) | 22.1 (21.7- 22.6) | 23.7 (23.2 - 24.2) | 22.0 (21.6 - 22.5) |

Note: The sensitivity analysis considered the same events as the main analysis, but added the admissions for which CAP was recorded as additional diagnosis and POA was coded as unknown or undetermined. Because the POA flag was implemented in Portugal only in 2013, 2010-2012 could not be included.

CI: confidence interval.
